# Investigation of Temperature Variations and Extreme Temperature Differences for the Corrugated Web Steel Beams under Solar Radiation

**DOI:** 10.3390/s22124557

**Published:** 2022-06-16

**Authors:** Shiji Huang, Chenzhi Cai, Yunfeng Zou, Xuhui He, Tieming Zhou

**Affiliations:** 1 School of Civil Engineering, Central South University, Changsha 410075, China; 194811084@csu.edu.cn (S.H.); yunfengzou@csu.edu.cn (Y.Z.); xuhuihe@csu.edu.cn (X.H.); tmzhou@csu.edu.cn (T.Z.); 2 National Engineering Research Center of High-Speed Railway Construction Technology, Central South University, Changsha 410075, China; 3 Hunan Provincial Key Laboratory for Disaster Prevention and Mitigation of Rail Transit Engineering Structures, Central South University, Changsha 410075, China

**Keywords:** steel beams, corrugated web, temperature distribution, experiment measurement, extreme value analysis

## Abstract

Due to the coupling impacts of solar radiation, wind, air temperature and other environmental parameters, the temperature field of steel structures is significantly non-uniform during their construction and service stages. Corrugated web steel beams have gained popularity in structural engineering during the last few decades, while their thermal actions are barely investigated. In this paper, both experimental and numerical investigations were conducted to reveal the non-uniform features and time variation of the corrugated web steel beams under various environmental conditions. The heat-transfer simulation model was established and verified using the experimental temperature data. Both the experiment and simulation results demonstrate that the steel beam has a complicated and non-uniform temperature field. Moreover, 2-year continuous numerical simulations of steel beams’ thermal actions regarding eight different cities were carried out to investigate the long-term temperature variations. Finally, based on the long-term simulation results and extreme value analysis (EVA), the representative values of steel beams’ daily temperature difference with a 50-year return period were determined. The extreme temperature difference of the steel beam in Harbin reached up to 46.9 °C, while the extreme temperature difference in Haikou was 28.8 °C. The extreme temperature difference is highly associated with the steel beam’s location and surrounding climate. Ideally, the outcomes will provide some contributions for the structural design regarding the corrugated web steel beam.

## 1. Introduction

Many steel spatial structures are inevitably exposed to complicated natural environments during their construction and service stages. The multifactor functioning of the structure’s surfaces leads to a significantly non-uniform temperature field [1,2]. The thermal stresses and deformations in these structures are larger and more complicated than those in structures which are not directly exposed to sunlight [3]. As structural deformation is prevented by redundant constraints, the thermal impact can sometimes become one of the key design loads. The steel member may fail or fracture considering the temperature change in addition to other loads, such as gravity and wind load. Moreover, the construction errors induced by the non-uniform temperature field may seriously affect the component assembly efficiency and structure closure.

Nowadays, corrugated web steel beams are widely applied in engineering structures due to their lightweight and superior carrying capacity [4,5]. The corrugated web steel beam’s temperature field can be more complicated and non-uniform on account of its complicated geometric construction [6]. Therefore, investigations of the non-uniform temperature field are of critical importance for improving their engineering application and ensuring the structural safety of corrugated web steel beams.

The temperature impacts on the steel spatial structures are generally expressed in the following two aspects: the mean temperature change and the non-uniform temperature distribution [2,7,8]. The mean temperature change of the steel structures can be considered as a slow and regular process, which is associated with the seasonal temperature variation. In the conventional design method, the mean temperature change is the main concern, while the non-uniform temperature distribution is always ignored. According to previously published research, the temperature field of the steel spatial steel structures is conspicuously non-uniform under the effect of surrounding thermal loads [9,10,11]. The non-uniform temperature field in the spatial structures certainly presents an evident influence on the structural behavior of the steel structures.

Plenty of researches have verified the existence of a non-uniform temperature field in the spatial structures. Zhao et al. [12] monitored the temperature distribution of a reticulated dome covered by an ETFE membrane. The experimental results indicated that the temperature field of the steel spatial structure is obviously non-uniform. Chen et al. [13] performed an experimental investigation on a beam string structure and its non-uniform features and temperature variations were analyzed on the basis of the experimental data. Meanwhile, a temperature simulation model for the beam string structure was established and verified by the measured temperature data. Chen et al. [14] further numerically studied the thermal behavior of a steel spatial truss structure. Based on the one-month simulation results, several suggestions regarding welding time and sequence were proposed for the closure construction of steel truss structures.

Furthermore, there are also some researchers devoting their attention to the temperature distribution in the steel members. Abid [15] measured the temperature distribution in a H-shaped steel beam during summer days and a simulation model was developed for thermal analysis. The impact of geometric parameters on the temperature distribution were analyzed on basis of the numerical simulation method. Liu et al. [16] carried out an experimental and numerical investigation on the temperature field of an H-shaped steel member. A simple method was developed for predicting the temperature distribution of steel beams. These studies indicate that the steel members also have non-uniform temperature distributions under solar radiation. Although the non-uniform temperature field exists in spatial structures or steel members, investigations with respect to the thermal actions of these steel structures are insufficient [17,18,19]. Moreover, there is no effective and accurate method by which to consider the thermal impact of surrounding environmental conditions on the steel structures.

This paper focuses on the non-uniform features and time variation of corrugated web steel beams under various environmental conditions. A corrugated web steel beam was fabricated and utilized for experimental investigation. The numerical simulation method was proposed for the thermal analysis and its accuracy was verified by the experimental temperature data. Then, 2-year continuous numerical simulations of steel beams’ thermal actions regarding eight different cities were carried out to investigate the long-term temperature variations. Finally, based on the long-term simulation results and extreme value analysis method, the representative values of steel beams’ daily temperature difference with a 50-year return period were determined.

## 2. Experimental Procedures

**Figure 1:**
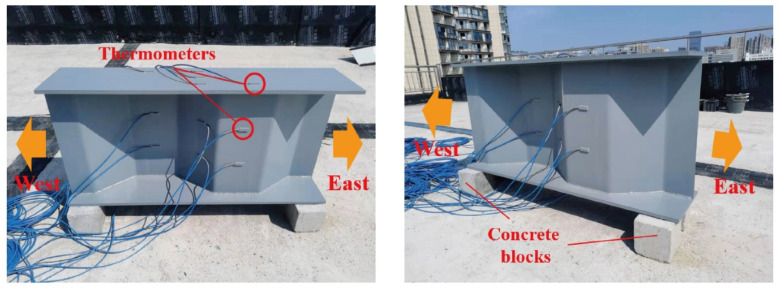
The photograph of the experimental steel beam.

The experimental work was carried out using a steel beam with a corrugated web which was assembled on the campus of Central South University at Latitude: 28°8′23″ N and Longitude: 112°59′10″ E, as shown in Figure 1. The experimental site was located on the rooftop of a six-story lab and surrounding buildings had no sheltering effects on the steel beam specimen. The longitudinal axis of the experimental specimen was placed in the East–West direction. The south side of the specimen was exposed to the sunlight during the daytime, while the north side of the specimen barely received solar radiation.

**Figure 2:**
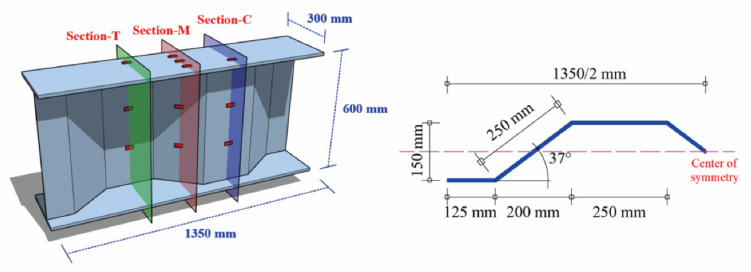
The detailed dimensions of the steel beam with corrugated web.

The experimental steel beam was fabricated with two 15 mm thick steel plates and one 9 mm thick corrugated steel plate. The detailed dimensions of the steel beam are provided in Figure 2. The length, width and height of the experimental steel beam were 1350 mm, 300 mm, and 600 mm, respectively, whereas the wave length, wave height, and bending angle of the corrugated steel web were 900 mm, 150 mm, and 37°, respectively.

**Figure 3:**
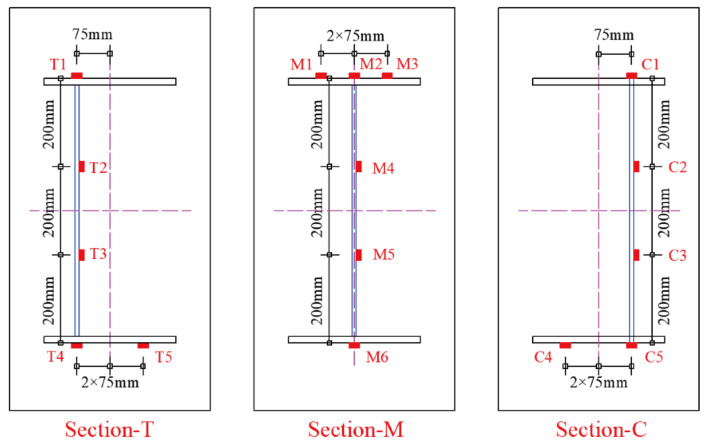
The detailed locations and serial numbers of thermometers.

A total of 16 PT1000 platinum resistance thermometers were installed on the surfaces of the steel beam for temperature measurement. These thermometers were arranged at three different cross-sections, as shown in Figure 2. The cross-sections at the crest and trough of the steel beam are, respectively, denoted as Section-C and Section-T, while the middle cross-section of the steel beam is denoted as Section-M. The detailed positions of these thermometers and their serial numbers are provided in Figure 3.

**Figure 4:**
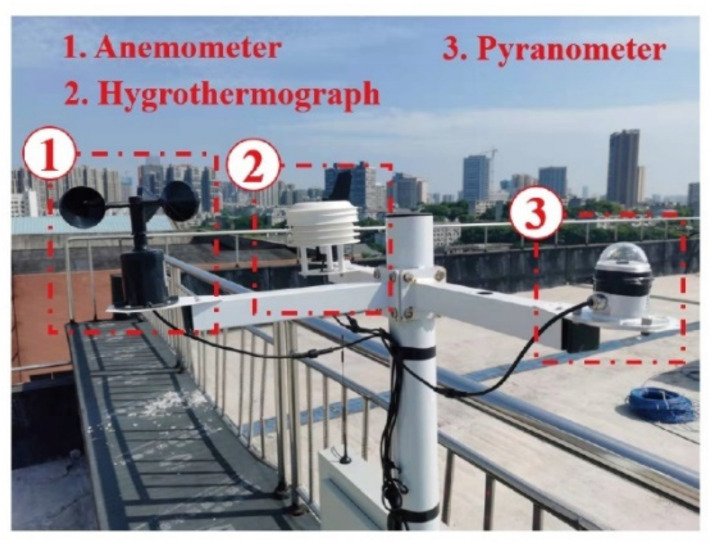
Small weather monitoring station.

The experimental scheme also included the measurement of surrounding climatic data. A small weather station was assembled at the experimental site for climatic data acquisition, as displayed in Figure 4. The anemometer, hygrothermograph, and pyranometer were applied to measure the wind speed, air temperature, and solar radiation intensity, respectively. The temperature of the steel beam and its surrounding climatic data 2343 synchronously recorded every 15 min from 17 August 2021 to 30 September 2021.

## 3. The Experimental Results

### 3.1. Air Temperature, Wind Speed, and Solar Radiation

**Figure 5:**
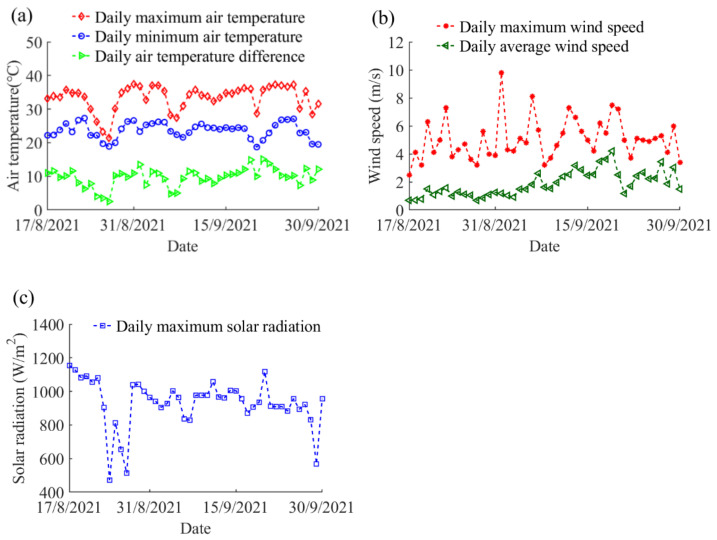
The recorded climatic data during the experimental period: (**a**) air temperature; (**b**) wind speed; and (**c**) solar radiation.

The temperature distribution of the steel beam highly correlated with the recorded climatic data. The air temperature is one of the main factors affecting the boundary thermal loading in terms of convection and long-wave radiation. Figure 5a illustrates the variations in the daily maximum and minimum air temperatures during the whole experimental period, as well as the daily air temperature differences. The highest air temperature reached 37.5 °C on 31 August 2021, while the lowest air temperature dropped to 18.7 °C on 20 September 2021. On the other hand, the maximum daily air temperature difference was 15.1 °C, which was recorded on 21 September 2021.

The wind speed contributes to a significant influence on the convection cooling process. The daily maximum and average wind speed along the complete experimental period are given in Figure 5b. The daily average wind speed ranged from 0.7 m/s to 4.2 m/s, while the highest wind speed reached 9.8 m/s on 1 September 2021. The solar radiation intensity dominates the heat flux from global solar radiation on the boundary surfaces. Figure 5c shows the daily maximum solar radiation intensity along the measurement peri-od. On sunny days, the daily maximum solar radiation intensity generally ranged from 900 to 1100 W/m^2^. The highest solar radiation intensity of 1155 W/m^2^ occurred on 17 August 2021.

### 3.2. Vertical Temperature Distributions

**Figure 6:**
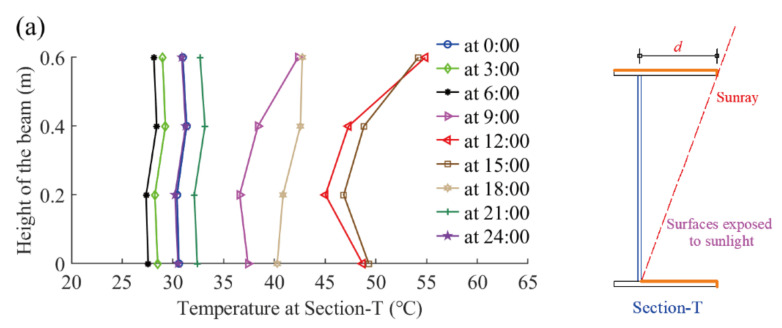

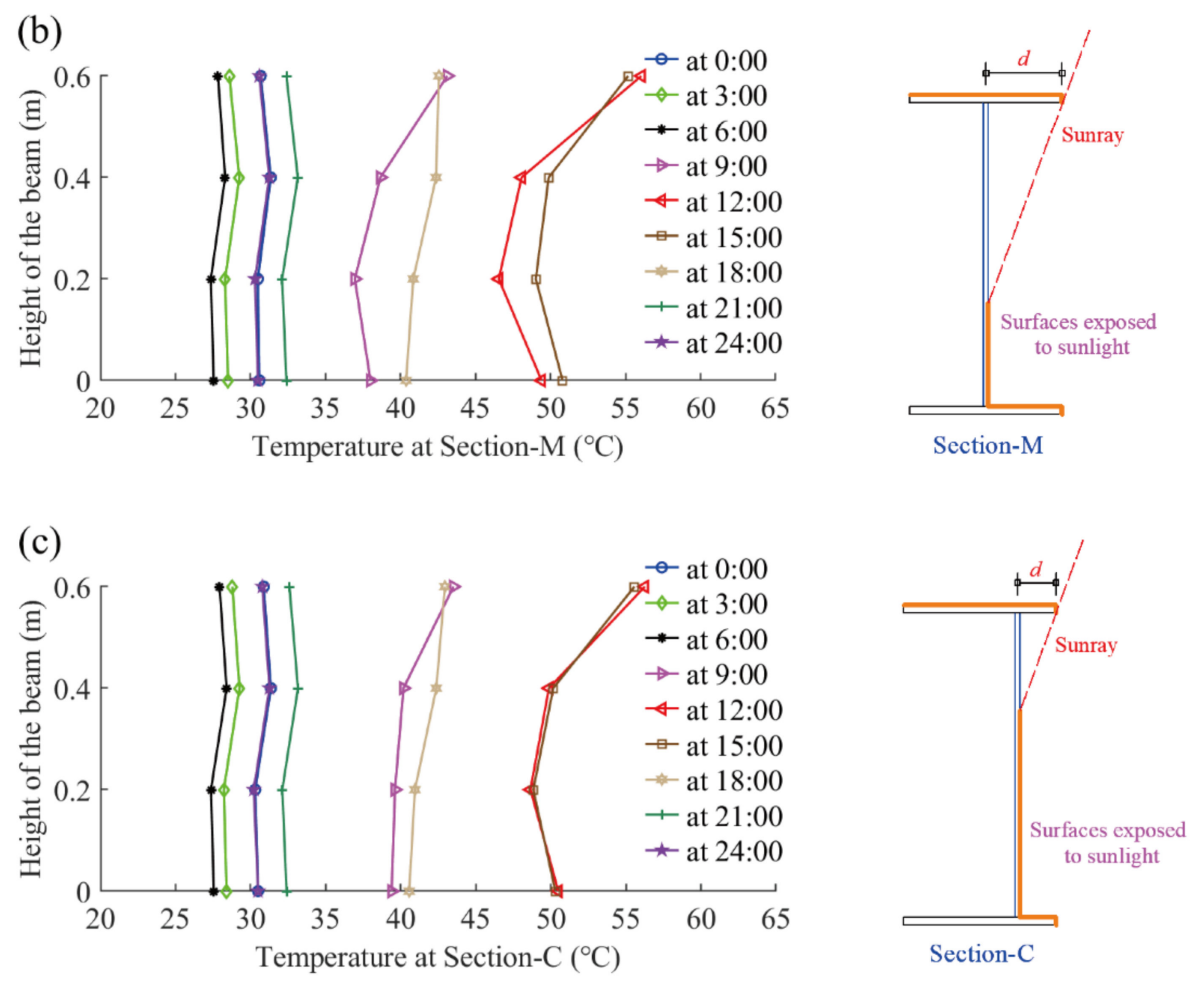
Vertical temperature distributions of the steel beam: (**a**) Section-T; (**b**) Section-M; and (**c**) Section-C.

In this section, the steel beam’s vertical temperature variation in a typical day is studied in detailed. The typical day was determined to be 31 August 2021 on which the highest sample temperature was recorded. The vertical temperature distributions at different times on the typical day are demonstrated in Figure 6. The thermal loading conditions on the steel beam’s surfaces are different among three cross-sections. The steel web surfaces at Section-T are shaded by the top flange in the daytime, while the steel web surfaces at Section-M and Section-C are directly exposed to the sunlight. This phenomenon brings about different temperature distributions among three sections. The magnitude of the steel web’s temperature in Section-C is the maximum, followed by that in Section m and Section-T, respectively. The maximum vertical temperature gradients at Section-T, Section-M, and Section-C are 10.2 °C, 10.1 °C, and 7.9 °C, respectively.

**Figure 7:**
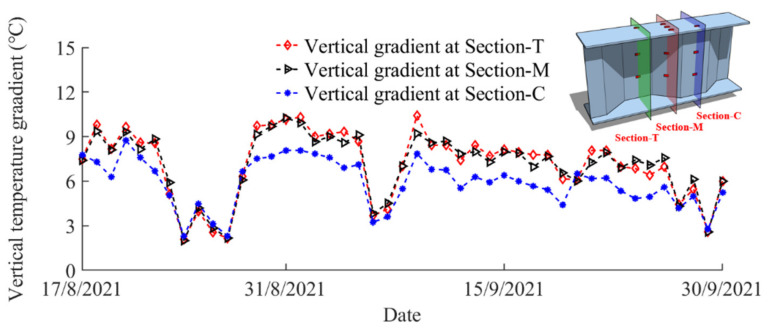
The daily maximum vertical temperature gradient at different sections.

The daily maximum vertical temperature gradients at Section-T, Section-M, and Section-C during the whole experimental period are presented in Figure 7. It can be observed that the daily maximum vertical temperature gradients at Section-T and Section m have the similar variation, while the vertical gradient at Section-C is evidently smaller than that at Section-T and Section-M. The daily maximum vertical temperature gradient at Section-T, Section-M, and Section-C reached up to 10.5 °C (on 9 September 2021), 10.2 °C (on 31 August 2021), and 8.8 °C (on 20 August 2021), respectively. The uniformity of the temperature field is conspicuous and it should be seriously considered in the construction and service stages of the steel structures.

### 3.3. Maximum and Minimum Temperatures

**Figure 8:**
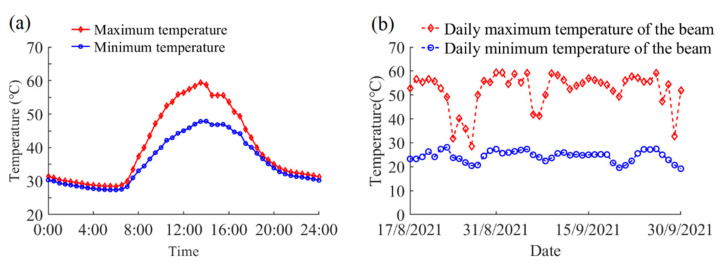
The daily maximum and minimum temperatures of the experimental steel beam: (**a**) on the typical day; (**b**) during the complete experiment period.

The steel beam’s maximum and minimum temperatures on the typical day are shown in Figure 8a. The temperature of the steel beam increased rapidly after sunrise and reached the peak value 59.4 °C at 13:30, while the minimum temperature of the steel beam was 27.4 °C, which was observed at nighttime. Figure 8b illustrates the recorded daily maximum and minimum temperatures during the complete experimental period. It can be observed that the variation trend of the daily maximum temperature is consistent with the daily maximum solar radiation. While the daily minimum temperature has the similar variation with the daily minimum air temperature. The recorded daily maximum temperature of the steel beam reached 59.4 °C on 31 August 2021, while the measured minimum temperature dropped to as low as 19.2 °C on 20 September 2021.

## 4. Finite Element Model of the Steel Beam

### 4.1. Basic Theory for Thermal Analysis

The heat conduction process inside the steel beam was dominated by the Fourier partial differential equation expressed as below [20]:

(1) $$k\left(\frac{\partial^{2}T}{\partial x^{2}}+\frac{\partial^{2}T}{\partial y^{2}}+\frac{\partial^{2}T}{\partial z^{2}}\right)=\rho c\frac{\partial T}{\partial t},$$

where *T* represents the temperature at any point in the steel beam, *t* refers to the time variable, *k* stands for the thermal conductivity coefficient, *ρ* stands for the density, and *c* is the specific heat. The initial temperature distribution *T_t_*_=0_ and the boundary thermal flux *q* of the exterior surfaces of the steel beam are given as:

(2) $$T_{t=0}=f\left(x,y,z\right)$$

(3) $$k\frac{\partial T}{\partial x}n_{x}+k\frac{\partial T}{\partial y}n_{y}+k\frac{\partial T}{\partial z}n_{z}+q=0,$$

where *f*(*x*,*y*,*z*) represents the beam’s temperature distribution at the starting time and the terms *n_x_*, *n_y_*, and *n_z_* stand for the direction cosines of the normal vectors. The total heat flux *q* on the boundary surfaces consists of the following four portions [21,22,23]: the convection heat flux *q_c_*, the total solar radiation heat flux *q_s_*, the long-wave radiation heat flux *q_l_*, and the mutual radiation heat flux *q_m_*.

The heat flux induced by convection can be evaluated with following equation:

(4) $$q_{c}=h_{c}\left(T_{s}-T_{a}\right),$$

where *h_c_* is the convection coefficient and *T_s_* and *T_a_* represent the temperatures of the beam’s surfaces and surrounding air, respectively. The convection coefficient is associated with the wind speed and surface roughness, which can be calculated with [16,24]:

(5) $$h_{c}=\sqrt{{\left[C_{t}{\left(T_{s}-T_{a}\right)}^{1/3}\right]}^{2}+{\left[aV^{b}\right]}^{2}},$$

where *V* represents the wind speed at standard conditions, *C_t_* is the turbulent natural convection constant and *a*, *b* are the constants.

The heat flux from solar radiation can be calculated as follows:

(6) $$q_{s}=\alpha I_{T}$$

where *α* is the absorptivity of the steel surfaces, and *I_T_* represents the total solar radiation intensity on each surface of the beam.

Based on the surface’s orientation and the solar position, the solar radiation intensity on an arbitrary surface can be calculated as follows [25,26,27]:

(7) $$I_{T}=I_{b}\left(\frac{\cos\theta }{\cos\theta_{z}}\right)+I_{d}\left(\frac{1+\cos\beta }{2}\right)+\rho \left(I_{b}+I_{d}\right)\left(\frac{1-\cos\beta }{2}\right),$$

where *I_b_* and *I_d_* stand for the direct and diffuse radiation intensity on a horizontal surface, respectively, *θ* represents the incident angle of the sunlight, *θ_z_* is the zenith angle, *β* represents the tilt angle of the surface, and *ρ* is the ground surface reflectance.

The long-wave radiation heat-transfer occurring between the beam’s surfaces and surrounding atmosphere can be evaluated by:

(8) $$q_{l}=\varepsilon C_{B}\left(T_{s}^{4}-T_{a}^{4}\right),$$

where *ε* is the emissivity of the steel surface and *C_B_* stands for the Stefan–Boltzmann constant.

The steel’s thermal properties applied in this paper are as follows [6]: thermal conductivity = 56 W/mK, density = 7850 kg/m^3^, and specific heat = 480 J/kgK. Besides, the steel surface’s absorptivity, emissivity, and ground reflectance values were set as 0.6, 0.8, and 0.2, respectively. All these values are available from previous studies. The above-mentioned boundary thermal loads were all considered and the recorded surrounding environmental data were inputted in the simulation model to calculate the heat flux on the boundary surfaces.

### 4.2. Finite Element Model

The thermal analysis of the corrugated web steel beam was performed using the Finite Element (FE) software COMSOL Multiphysics 5.5 in this paper. The inside heat conduction process and the boundary conditions of the exterior surfaces were realized using different built-in interfaces in COMSOL [28]. The Heat Transfer in Solids interface can simulate the heat conduction inside the structure and convection on the boundary surfaces, while the Surface-to-Surface Radiation interface can model the radiative heat-transfer process. The mutual radiation and shade effects were evaluated using the hemicube method in this study. This method can calculate the angle coefficient between two arbitrary surfaces and the radiation energy percentage that emits from a surface to another surface [29]. Besides, the sun position that rotates with time can be determined using the solar radiation in COMSOL based on the structure’s location, time zone, date, and local time.

**Figure 9:**
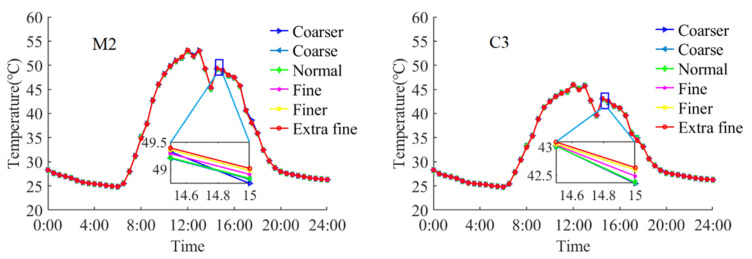
The temperature variation of M2 and C3 for different mesh sizes.

The FE model is meshed by the physics-controlled mesh in COMSOL which includes nine different precision grades from extremely coarse to extremely fine. The finer mesh size is adopted in the FE model and it is proved to have sufficient accuracy for the thermal analysis of steel beams according to previous researches [6,15]. The FE model contains a total of 56,952 tetrahedra elements and 38,014 triangular elements. To ensure the accuracy and efficiency of the FE model, a convergence analysis was conducted by six analysis runs with different mesh sizes. The recorded environmental data in 13 September 2021 were inputted in the FE model. Figure 9 presents the temperature variation of two measurement points (M2 and C3) with respect to different mesh sizes. The maximum error is less than 0.3 °C. Thus, the mesh size used in the FE model can satisfy the requirement of convergence.

The starting time was set to midnight when the temperature was approximately uniform through the steel beam. The mean temperature of all the thermometers at midnight was applied as the initial temperature of the FE model. The time step of the thermal analysis was set to be 0.25 h.

### 4.3. Model Validation

**Figure 10:**
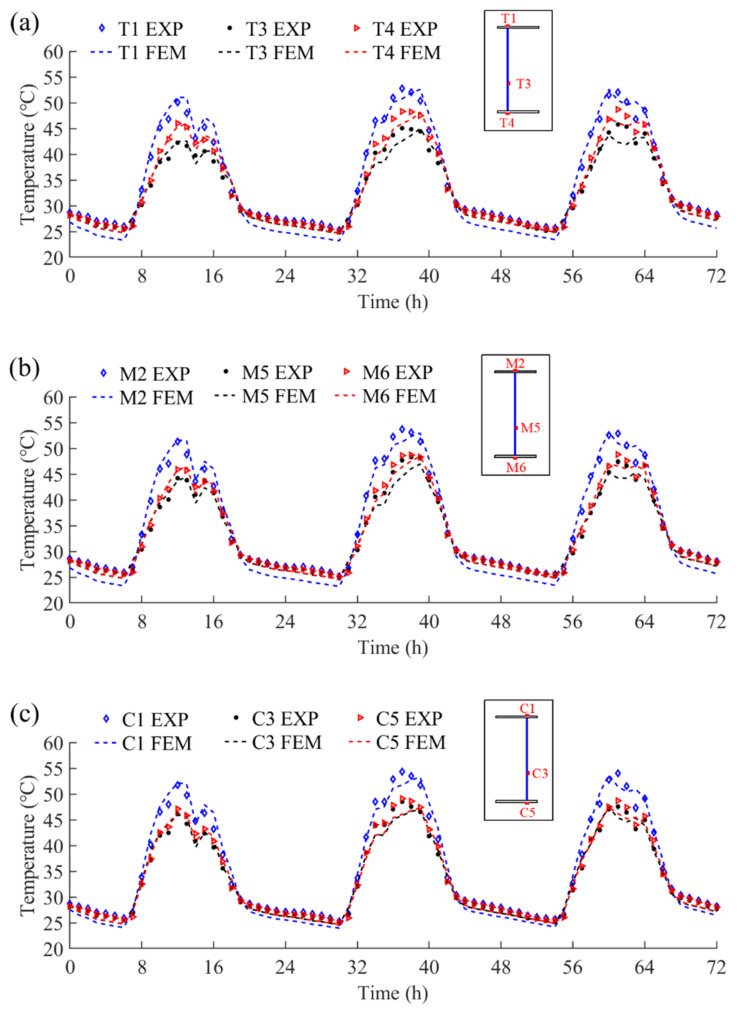
The comparison of experimental and simulated temperatures on the selected days: (**a**) measurement points at Section-T; (**b**) measurement points at Section-M; and (**c**) measurement points at Section-C.

Three-day experimental temperatures (from 13 September 2021 to 15 September 2021) were used to validate the FE model. These days were continuous sunny days. Thus, the steel beam’s temperature distribution characteristics should not be affected by weather variations. Measurement points at Section-T (T1, T3, and T4), Section-M (M2, M5, and M6), and Section-C (C1, C3, and C5) were selected to compare the experimental and the simulated temperatures, as illustrated in Figure 10. The EXP and FEM in the figures stand for the experimental temperatures and the finite element simulated temperatures, respectively. It can be observed that the variations and the magnitudes of the finite element simulated temperatures match well with the experimental temperatures.

Two statistical indexes were introduced to investigate the degree of agreement between the experimental and the simulated temperatures. The first is the Maximum Absolute Error (MAE), which represents the maximum absolute difference between the experimental and the simulated temperatures at each measurement point during the selected days. The second is the Average Absolute Error (AAE), which refers to the sum of the absolute differences between the experimental and the finite element simulated temperature divided by the total number of temperature points. The MAE and AAE can be calculated with the following equations:

(9) $$\mathrm{MAE}=\max\left(T_{EXP}-T_{FEM}\right),$$

(10) $$\mathrm{AAE}=\frac{\Sigma \left|T_{EXP}-T_{FEM}\right|}{m}$$

where *T_EXP_* is the experimental temperature, *T_FEM_* represents the finite element simulated temperatures, and *m* is the total number of temperature points.

The MAE and AAE between the simulated and experimental temperatures are illustrated in Table 1. The MAE of the thermometers located at Section-T, Section-M, and Section-C are 3.5 °C, 3.8 °C, and 4.1 °C, respectively. On the other hand, the AAE of the thermometers located at Section-T, Section-M, and Section-C are 1.1 °C, 1.1 °C, and 1.0 °C, respectively. All these errors are within the acceptable range. Therefore, the developed FE simulation model provides sufficient accuracy to evaluate the temperature variations in the steel beam under solar radiation.

## 5. Long-Term Environmental Data

### Environmental Data

**Figure 11:**
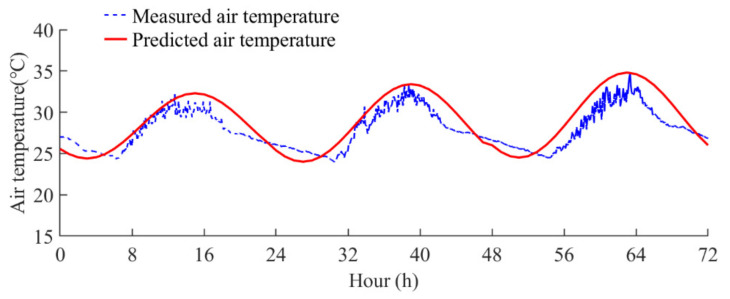
Comparison of the predicted and measured air temperatures.

The thermal analysis of the steel beam requires time-dependent environmental data. The variations in air temperature and solar radiation were regular and predictable. In the current study, empirical equations were adopted to generate the required time-dependent air temperature and solar radiation. The variation of the wind speed was irregular and the daily mean wind speed was adopted as the input data. For the variations in air temperature, the sinusoidal Kreith and Kreider equation was used to calculate the temporal variations in air temperature [30]:

(11) $$T_{a}\left(t\right)=\frac{\left(T_{\max}+T_{\min}\right)}{2}+\frac{\left(T_{\max}-T_{\min}\right)}{2}\sin\frac{\pi \left(t-9\right)}{12},$$

where *T_max_* and *T_min_* are the daily maximum and minimum air temperatures, respectively. Figure 11 shows the air temperatures predicted using Equation (11) and the measured air temperatures from 13 September 2021 to 15 September 2021. It can be observed that the predicted air temperatures are in good agreement with the measured air temperatures. Thus, the Kreith and Kreider equation presents sufficient accuracy for describing the variations in air temperature.

**Figure 12:**
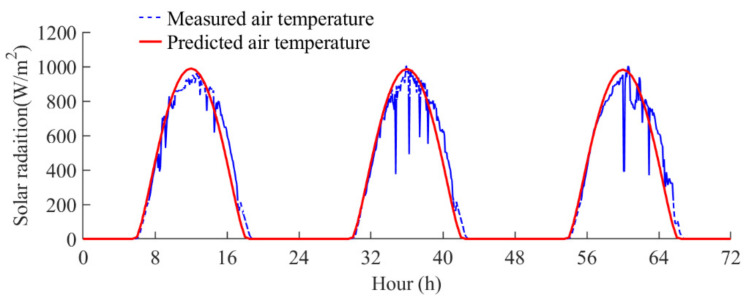
Comparison of the predicted and measured solar radiation.

The surface solar radiation is dependent on the solar constant *I_sc_* and the transmission in the atmosphere. The temporal variations of the solar radiation can be calculated by the empirical equations provided below [21,31,32]:

(12) $$I_{b}=k_{b}I_{sc}\left(1+0.033\cos\frac{360n}{365}\right)\sin h,$$

(13) $$I_{d}=k_{d}I_{sc}\left(1+0.033\cos\frac{360n}{365}\right)\sin h,$$

where *I_b_* and *I_d_* represent the direct radiation and diffuse radiation on a horizontal plane, respectively, *n* is the day of the year (which ranges from 1 to 365), and *h* is the solar altitude angle. *k_b_* and *k_d_* are the transmission coefficients regarding direct radiation and diffuse radiation, respectively. The transmission coefficients can reflect the attenuation of solar radiation when it passes through the atmosphere. The coefficients can be calculated by [33,34]:

(14) $$k_{b}=0.9^{m\cdot t_{u}},$$

(15) $$k_{d}=0.2710-0.2939k_{b},$$

(16) $$m=\frac{k_{a}}{\sin\left(h+5{}^{\circ}\right)},$$

where *k_a_* represents the relative atmospheric pressure, and *t_u_* represents the Linke turbidity coefficient, which is an indicator of atmospheric opacity. The predicted solar radiation was compared with the measured solar radiation from 13 September 2021 to 15 September 2021, as presented in Figure 12. As is presented in the figure, the predicted solar radiation agrees well with the measured solar radiation. Thus, these empirical equations were adopted to predict the time-varying solar radiation.

**Figure 13:**
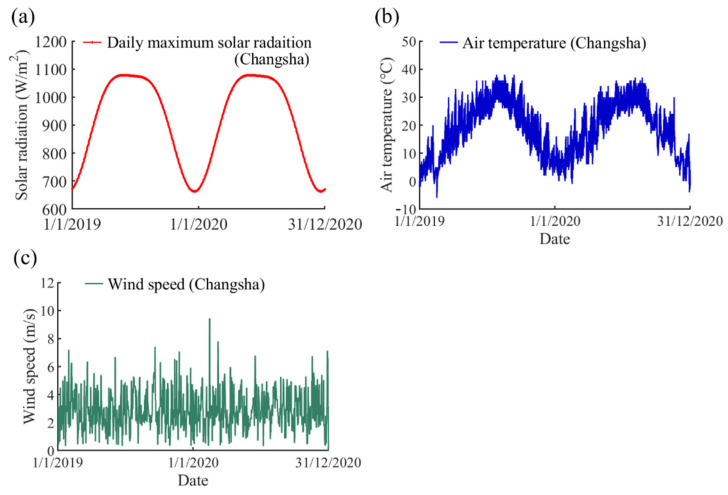
The long-term climatic parameter variation in Changsha: (**a**) daily maximum solar radiation; (**b**) air temperature; and (**c**) wind speed.

The long-term variations in climatic parameters in eight different cities (including Harbin, Changsha, Jinan, Shanghai, Haikou, Kunming, Naqu, and Turpan) from 1 January 2019 to 31 December 2020 were obtained on the basis of the above-mentioned empirical equations. The detailed locations and climatic types of these cities are given in Table 2. These cities are located in different climatic regions and they can reflect the climatic characteristics of most parts of China. The obtained climatic data of eight cities were inputted in the simulation model to investigate the temperature variations of steel beams in different climatic regions. The variations of daily maximum solar radiation, air temperature, and wind speed in Changsha were selected as examples and are presented in Figure 13.

## 6. Results of the Long-Term Simulation

**Figure 14:**
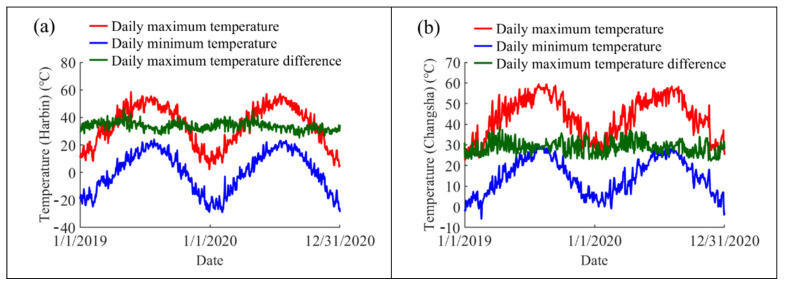

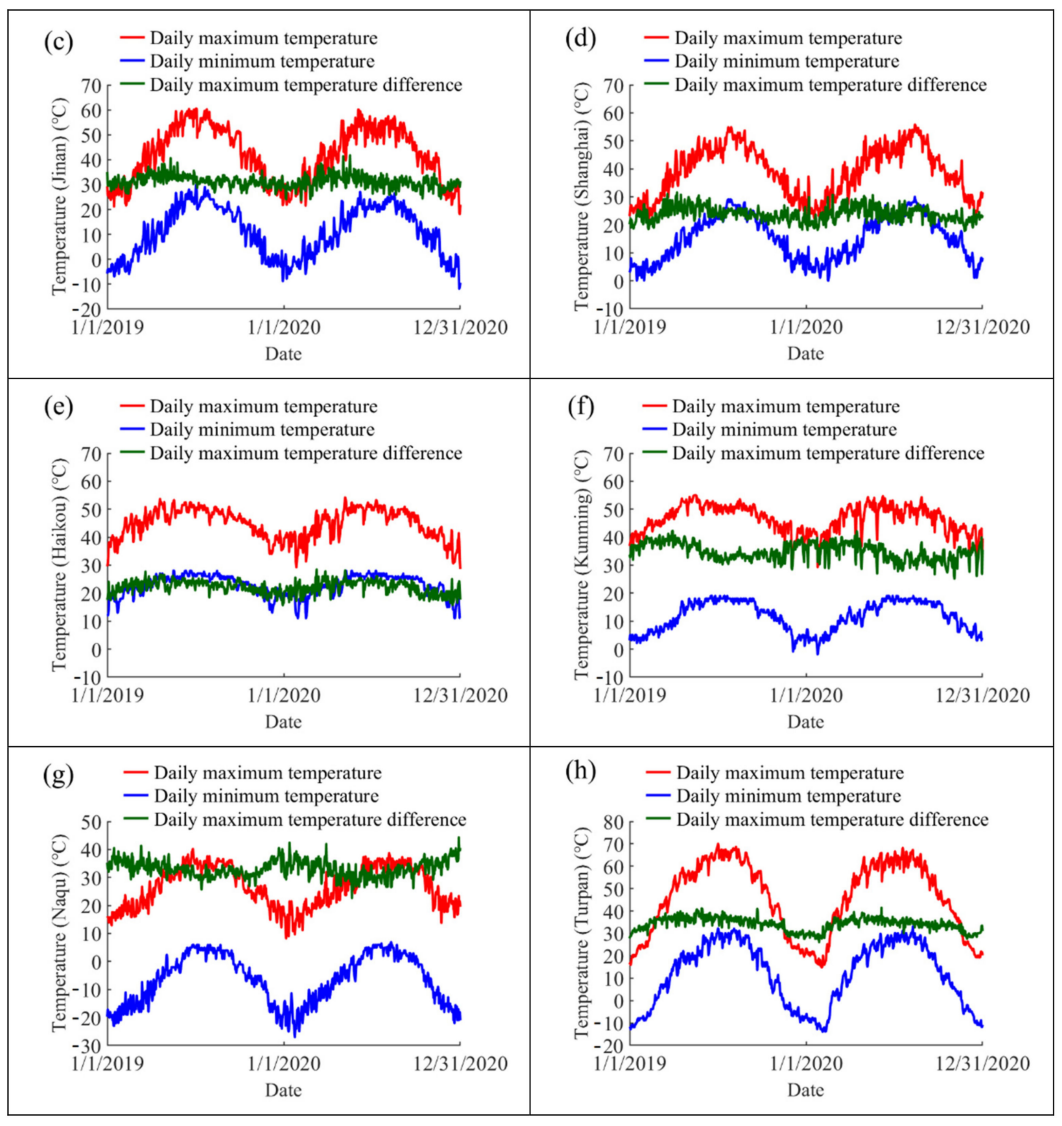
Daily maximum temperatures, daily minimum temperatures, and daily maximum temperature differences of steel beams in different cities: (**a**) Harbin; (**b**) Changsha; (**c**) Jinan; (**d**) Shanghai; (**e**) Haikou; (**f**) Kunming; (**g**) Naqu; and (**h**) Turpan.

Three indexes (including the daily maximum temperatures *V_max_*, the daily minimum temperatures *V_min_*, and the daily maximum temperature differences *V_diff_*) were introduced to describe the thermal actions in the steel beam. The daily maximum and minimum temperatures were, respectively, defined as the highest and the lowest temperatures that the steel beam is able to reach under the impact of solar radiation, whereas the daily maximum temperature difference refers to the difference value of the daily maximum and minimum temperatures. The variations of these indexes during the whole simulation period are presented in Figure 14. As is presented in the figures, both the daily maximum and minimum temperatures exhibit remarkable seasonal variation across a period of one year, while the daily maximum temperature difference demonstrates an irregular annual distribution.

The extreme values of the steel beam’s maximum temperature *V_max_*, minimum temperature *V_min_*, and maximum daily temperature difference *V_diff_* during the complete simulation period are given in Table 3. The maximum value of *V_max_* in Turpan reached up to 70.1 °C on 4 July 2019, while the minimum value of *V_min_* in Naqu dropped to as low as −27.0 °C on 24 January 2020. The maximum annual temperature changes of the steel beams in Harbin, Changsha, Jinan, Shanghai, Haikou, Kunming, Naqu, and Turpan are 87.5 °C, 65.3 °C, 72.4 °C, 55.9 °C, 65.2 °C, 67.2 °C, 65 °C, and 84.0 °C, respectively. Besides, the daily maximum temperature changes of steel beams in these cities can reach 46.7 °C, 38.9 °C, 41.8 °C, 31.6 °C, 28.5 °C, 42.3 °C, 44.3 °C, and 41.5 °C, respectively. These annual and daily temperature differences may result in excessive stresses and deformations, which should be seriously considered in the design phase of steel structures. Moreover, the temperature indexes of the steel beam are highly correlated with the steel beam’s location and climatic type.

## 7. Extreme Value Analysis for Thermal Gradient

The daily temperature difference is an important temperature index for evaluating the thermal stresses and deformations of the steel beam. From a statistical point of view, the extreme daily temperature differences during the beam’s whole life cycle may exceed the simulated maximum temperature differences. In this section, the steel beam’s extreme daily temperature difference is determined on the basis of the probability theory considering a return period of 50 years. The probability *P* for the temperature gradients exceeding the extreme value can be calculated as [21,35]:

(17) $$P=\frac{1}{50\times N},$$

where *N* represents the amount of data in a full year, which is equal to 365 in this study.

### 7.1. Extreme Value Analysis

The extreme value analysis (EVA) method is widely applied in structural engineering to determine the representative value of environmental loads (such as the wind load and the earthquake load). Therefore, the generalized extreme value (GEV) distribution is one of the most significant analysis methods in EVA, which was adopted in the current study for determining the extreme thermal gradients of steel beams. The cumulative distribution function (CDF) of the GEV distribution is given as [35,36]:

CDF:

(18) $$H\left(TD;\mu ,\sigma ,\xi \right)=\exp\left[-{\left(1+\xi \frac{TD-\mu }{\sigma }\right)}^{-1/\xi }\right]1+\xi \frac{TD-\mu }{\sigma }>0,$$

where *TD* is the vertical temperature gradient, and *μ*, *σ*, and *ξ* represent the location parameter, scale parameter, and shape parameter, respectively. The type of limiting distribution is associated with the magnitude of the shape parameter *ξ*. When *ξ* = 0, *ξ* > 0, and *ξ* < 0, the GEV distribution corresponds to the Gumbel distribution, Fréchet distribution, and Weibull distribution, respectively. Based on the exceedance probability *P* and the probability density function (PDF) of GEV distribution, the extreme thermal gradient *TD_e_* can be determined as follows:

(19) $$f\left(TD;\mu ,\sigma ,\xi \right)=\frac{1}{\sigma }\left[-{\left(1+\xi \frac{TD-\mu }{\sigma }\right)}^{-1/\xi }\right]{\left(1+\xi \frac{TD-\mu }{\sigma }\right)}^{-\left(1+1/\xi \right)}1+\xi \frac{TD-\mu }{\sigma }>0,$$

(20) $$P=\int_{TD_{e}}^{+\infty }f\left(TD;\mu ,\sigma ,\xi \right)dTD.$$

### 7.2. GEV Distribution and Extreme Temperature Difference

**Figure 15:**
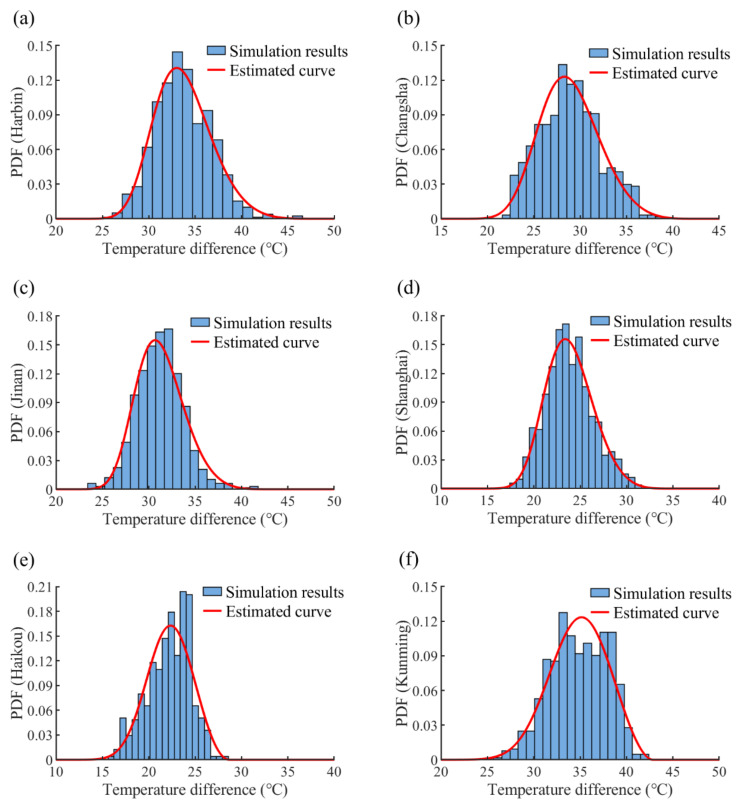

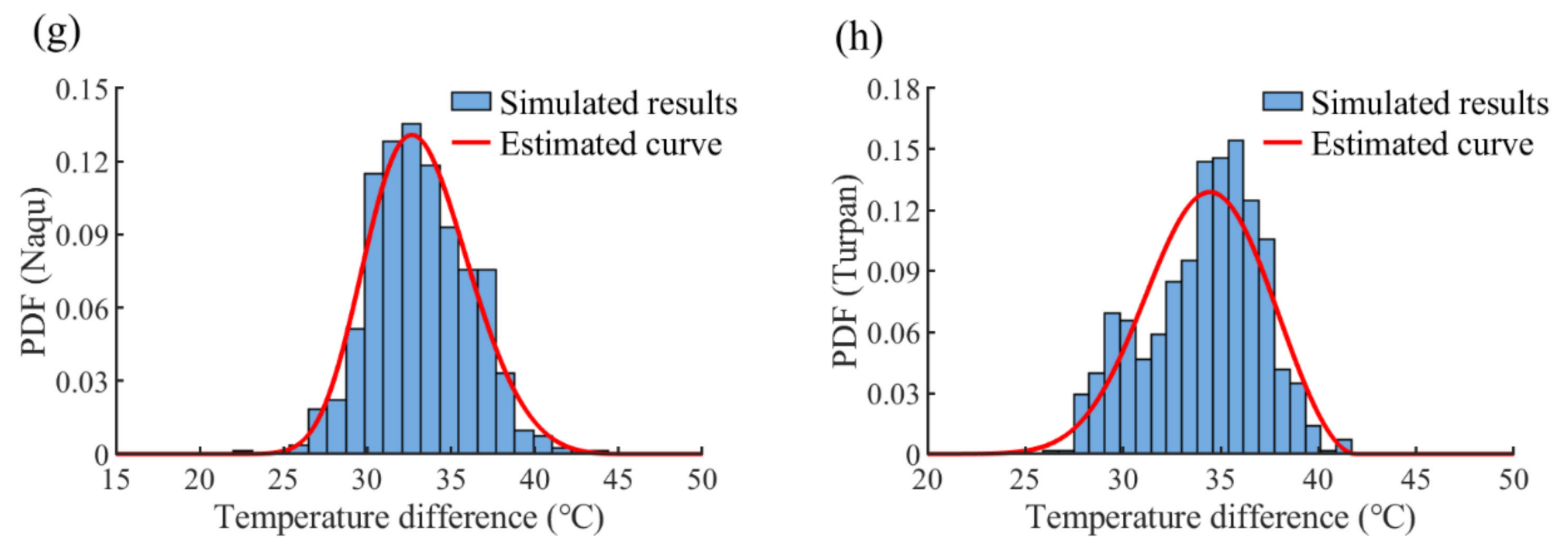
Frequency histogram and fitted PDF curves regarding different cities: (**a**) Harbin; (**b**) Changsha; (**c**) Jinan; (**d**) Shanghai; (**e**) Haikou; (**f**) Kunming; (**g**) Naqu; and (**h**) Turpan.

The daily temperature differences of steel beams in different locations from 1 January 2019 to 31 December 2020 were taken as samples for the extreme value analysis. The parameters of the GEV distribution were determined using curve fitting. The frequency histograms and fitted PDF curves are demonstrated in Figure 15. The parameters of fitted GEV distributions and the representative values of daily temperature differences with a 50-year return period are outlined in Table 4. Thermal actions always follow the Weibull distribution or the Gumbel distribution according to several previous studies. In the current investigation, all the fitted shape parameters are less than zero, which indicates that all the curves follow the Weibull distribution. Based on the fitted PDF curves and Equation (20), the representative values of steel beams’ daily temperature differences in Harbin, Changsha, Jinan, Shanghai, Haikou, Kunming, Naqu, and Turpan are 46.9 °C, 40.8 °C, 41.9 °C, 33.3 °C, 28.8 °C, 42.6 °C, 44.4 °C, and 41.7 °C, respectively.

In practical steel structures, the steel members have different orientations and inclinations. The steel members present different thermal behaviors under the impact of various environmental conditions. Furthermore, the temperature distributions of steel structures are highly correlated with the steel beams’ locations and climatic types. All these factors should be taken into consideration in the accurate determination of extreme thermal loads on practical steel structures. Hopefully, the outcomes can provide some references for the structural design regarding corrugated web steel beams.

## 8. Conclusions

In this investigation, an experimental study was performed to reveal the non-uniform features and time variation of the corrugated web steel beams under various environmental conditions. The simulation method was developed for the thermal analysis and its accuracy was verified with the recorded temperature data. The thermal actions of steel beams located in eight different cities were simulated continuously for 2 years. Based on the numerical results and extreme value analysis, the representative values of steel beams’ daily temperature difference were determined. Several conclusions were reached, and are summarized as follows:

(1) The selected three cross-sections were subjected to different thermal loadings, demonstrating different temperature distributions. The magnitude of the web’s temperature at Section-C was highest, followed by that at Section-M and Section-T, respectively. The maximum vertical temperature gradient at Section-T, Section-M, and Section-C reached up to 10.5 °C, 10.2 °C, and 8.8 °C, respectively. The experimental results demonstrate that the steel beam has a complicated and non-uniform temperature field.
(2) The numerical simulation method was proposed for the thermal analysis and its accuracy was verified by the experimental temperature data. The MAE of the thermometers located at Section-T, Section-M, and Section-C are 3.5 °C, 3.8 °C, and 4.1 °C, respectively. On the other hand, the AAE of the thermometers located at Section-T, Section-M, and Section-C are 1.1 °C, 1.1 °C, and 1.0 °C, respectively.
(3) The long-term variations of steel beams’ daily maximum temperature, daily minimum temperature, and the daily temperature difference regarding different regions were provided. The extreme value of the daily maximum temperature of the steel beam in Turpan reached up to 70.1 °C on 4 July 2019, while the extreme value of the daily minimum temperature of the steel beam in Naqu dropped to as low as −27.0 °C on 24 January 2020. The extreme daily temperature changes of the steel beam in Harbin reached up to 46.7 °C.
(4) The representative values of steel beams’ daily temperature difference with a 50-year return period were determined with an extreme value analysis. All the daily temperature differences in relation to the eight cities studied fit well with the Weibull distribution. The representative values of steel beams’ daily temperature differences in Harbin, Changsha, Jinan, Shanghai, Haikou, Kunming, Naqu, and Turpan are 46.9 °C, 40.8 °C, 41.9 °C, 33.3 °C, 28.8 °C, 42.6 °C, 44.4 °C, and 41.7 °C, respectively.

## Figures and Tables

**Table 1:** MAE and AAE between the simulated and experimental temperatures.

Thermometer Group	Serial Number	MAE (°C)	AAE (°C)
Section-T	T1	3.5	1.6
	T3	3.4	0.9
	T4	2.8	0.9
Section-M	M2	3.8	1.5
	M5	3.2	1.0
	M6	2.3	0.8
Section-C	C1	4.1	1.4
	C3	2.7	0.9
	C5	3.1	0.8

**Table 2:** Locations and climatic types of the selected cities.

Serial Number	City	Latitude	Longitude	Climatic Type
City I	Harbin	45°44′ N	126°36′ E	Temperate monsoon climate
City II	Changsha	28°08′ N	112°59′ N	Subtropical monsoon climate
City III	Jinan	36°40′ N	117°00′ N	Temperate monsoon climate
City IV	Shanghai	31°14′ N	121°28′ N	Subtropical monsoon climate
City V	Haikou	20°02′ N	110°20′ N	Tropical monsoon climate
City VI	Kunming	25°02′ N	102°43′ N	Subtropical plateau monsoon climate
City VII	Naqu	31°29′ N	92°04′ N	Plateau mountain climate
City VIII	Turpan	42°55′ N	89°12′ N	Temperate continental climate

**Table 3:** Extreme values of steel beam’s *V_max_*, *V_min_*, and *V_diff_* during the whole simulation period.

City	Maximum Value of *V_max_*		Minimum Value of *V_min_*		Maximum Value of *V_diff_*	
	Value	Date	Value	Date	Value	Date
Harbin	58.6 °C	24 May 2019	−28.9 °C	5 February 2020	46.7 °C	4 May 2019
Changsha	59.5 °C	19 August 2019	−5.8 °C	17 February 2019	38.9 °C	9 April 2019
Jinan	60.6 °C	4 July 2019	−11.8 °C	29 December 2020	41.8 °C	17 May 2020
Shanghai	55.8 °C	13 August 2020	−0.1 °C	16 February 2020	31.6 °C	9 April 2019
Haikou	54.2 °C	17 May 2020	11.0 °C	31 December 2020	28.5 °C	9 March 2020
Kunming	55.2 °C	16 May 2019	−1.9 °C	25 January 2020	42.3 °C	31 March 2019
Naqu	40.2 °C	27 June 2019	−27.0 °C	24 January 2020	44.3 °C	28 December 2020
Turpan	70.1 °C	3 July 2019	−13.9 °C	4 February 2020	41.5 °C	5 June 2020

**Table 4:** Parameters of fitted PDF curves and the representative values of daily temperature differences.

City	Type of Distribution	Shape Parameter (*ξ*)	Shape Parameter (*σ*)	Shape Parameter (*μ*)	Representative Value
Harbin	Weibull	−0.1693	2.8601	32.4923	46.9 °C
Changsha	Weibull	−0.1983	3.0534	27.6373	40.8 °C
Jinan	Weibull	−0.1762	2.4145	30.2210	41.9 °C
Shanghai	Weibull	−0.1970	2.4095	22.8696	33.3 °C
Haikou	Weibull	−0.3310	2.4055	21.4412	28.8 °C
Kunming	Weibull	−0.3570	3.2066	33.8427	42.6 °C
Naqu	Weibull	−0.2039	2.8739	32.0181	44.4 °C
Turpan	Weibull	−0.3533	3.0652	33.2078	41.7 °C

## Data Availability

Not applicable.
